# Informal and Formal Peer Teaching in the Medical School Ecosystem: Perspectives From a Student-Teacher Team

**DOI:** 10.2196/21869

**Published:** 2020-11-23

**Authors:** Anson Hei Ka Tong, Christopher See

**Affiliations:** 1 Faculty of Medicine The Chinese University of Hong Kong Hong Kong China (Hong Kong); 2 School of Biomedical Sciences Faculty of Medicine The Chinese University of Hong Kong Hong Kong China (Hong Kong)

**Keywords:** Peer learning, medical education, peer teaching, peer-led learning, peer, education

## Abstract

These personal views, drawn from the experiences of a medical student and a medical school lecturer, advocate caution of the current trend for formal adoption of peer teaching into medical school curricula. Using a metaphor from physics, we highlight the need for cautious deeper exploration of the informal world of peer-teaching in medical schools, which is a complex part of the educational ecosystem, prior to incorporating such activities into faculty-led initiatives. We support a measured approach to the introduction of compulsory peer-teaching activities given the recognized theoretical and pedagogical benefits.

## Introduction

In physics, the observer effect describes a situation involving a subatomic particle such as an electron, which has a certain momentum and position [1]. If one makes an attempt to measure the momentum of the electron, the very act of doing so will affect its position. Therefore, it is not possible to directly instrument such particles without altering the system that one wishes to observe.

Many peer-to-peer teaching moments in medical education might similarly be thought to exist in a kind of subatomic plane, or at least in one that is not always visible to faculty members. From the perspectives of a medical student and lecturer, we set out the impressive range of peer-led teaching that occurs in informal settings. We recognize the potential benefits of peer-led teaching in either informal or formal settings, including improved social and cognitive congruence between peer-teachers and students [2] and improved communication skills and teaching ability for participants [3]; however, we make the case that, despite these benefits, instrumenting the existing ecosystem of informal peer-teaching activities and incorporating them formally into faculty practices and curricula would be difficult to achieve without altering them fundamentally. The current trend in medical education literature of constructing formal peer-led medical education interventions and comparing improvements [4] is exactly the kind of instrumentation that should be considered carefully.

## A Student’s Perspective

Upon entering my university’s medicine course, I was randomly assigned by the faculty into a *small group* consisting of around 15 students. Initially, these groups were designed to serve a social rather than academic purpose. But what started off as a mere attempt by the faculty to promote a friendly setting for students slowly developed into a superb environment to cultivate student-initiated peer education.

The beauty of this small group system is the freedom to coordinate peer education both horizontally, allowing students of the same year to learn from one another, and vertically, with senior students teaching junior students. It has been a tradition for seniors to give advice and resources to juniors in the same small group; some seniors may even initiate study sessions. When I was in year 2 studying anatomy of the thorax and abdomen, a couple of year-3 students kindly organized such a study session for our group. They broke down complex concepts into simple language, arranged tedious details into mnemonics, and gave advice on how to order study topics when revising.

When I encountered the widely feared anatomy of the pelvis, there were a series of files created by a senior student being circulated within our class. Expecting to hear the familiar groans that come with the end of every perplexing pelvis lecture, I was surprised to instead hear everyone gushing over how amazing these materials were. Upon observation, these peer-made documents were excellent, simply because they were specifically curated for us, our curriculum, our examination, and our way of understanding. These beautifully color-coded notes spotlighted the most important concepts to know and included links to external videos or reference websites, hand-drawn diagrams, exam tips, and more.

In this curious underworld of peer education, learning comes in many forms—it is this multifaceted nature of peer education that demonstrates the imperceptible range of student learning practices. Yet, this closely networked form of peer education is not simply another study method. From a learner’s perspective, both formal and informal education serve key functions in the learning experience as a whole, forming what, I believe, is a symbiotic relationship between teaching from the official curriculum and student-led teaching.

To me, informal peer-education can provide additional learning experiences that formal education cannot provide. In addition to benefits such as higher engagement levels in smaller, casual environments, my experience with peer education is particularly treasured because I knew that my peer teacher had experiences similar to my own. I found it easier to digest certain explanations when presented in simple Cantonese with relatable analogies, compared to those presented in scholastic, jargon-heavy English. I felt that, in a very visceral way, I had a safety net for formal education in the form of this peer education network.

There is no single textbook able to provide all the information needed for a surgeon to practice well, and similarly, any one lecturer's words alone are not sufficient to provide a student with a well-rounded understanding of a topic—this is a gap in formal education that informal education can fill.

## A Teacher’s Perspective

Several vivid embarrassments as a lecturer have given me limited insight into the invisible role of peer teaching. After making a key point in a lecture, imagine seeing a pair of students chatting in the third row. I was rather displeased at this, and politely but firmly asked what private discussion was so important. With very red faces, the students were not forthcoming, so I went over to their seats. I was astonished to find a video of Michael Jackson doing his famous Moonwalk, his feet perfectly alternating in plantarflexion and dorsiflexion as I had described. “Is this what you mean?” one asked. It was my turn to turn extremely red. In my teaching, I had chosen a reference that was as iconic as I could imagine, but to 18-year-old students in Hong Kong, it was as alien as could be. Surely a peer would be able to communicate this point in a more culturally appropriate manner.

I was also astonished to discover that an alternative version of my lecture notes was in circulation. Senior students had taken to the PowerPoint slides with highlighter and pen, inserting English explanations and Cantonese characters, drawings, internet links, emojis, and practice questions. My lecture now lives in a student-only accessible cloud, together with edited versions of every other lecture in the course in a file known evocatively as the “God Disk.” With such a title, its significance to the student body may be hard to overestimate.

I was both offended and relieved at this discovery. Peer teaching in this form, it seems to me, was a kind of calibration and curation by students for students. Looking around further, informal peer teaching seemed to manifest in so many forms: microteaching moments between one student asking a question of another in the library, partners stalking a practical laboratory and solving problems in lockstep, or seniors and peers from school alumni groups, church groups, and beyond providing informal sharing. Peer teaching can range from the tiny to the enormous. It is my distinct feeling that I have just scratched the surface of what really goes on in a medical student’s education.

I have had other encounters where my experience in teaching was no match for the clarity of a peer-teaching moment. (Who knew that a macula hole in the retina looks like a specific kind of local dim sum known as a *Siu Mai*?) At times, I wondered if my role could be replaced by a well-trained army of peer teachers. On the other hand, students are not able to draw on clinical experiences of the emergency department late at night or of variant and unusual cadavers to illustrate learning points. A complex relationship exists where teacher-led and peer-led teaching together lay the path for the learning journey of medical school.

## Considerations For Educators

Medical education literature demonstrates a proliferation of peer-led teaching studies of varying sizes and designs [5,6]. Medical faculty members increasingly recognize the benefits of peer teaching, such as creating a safe learning space, teaching at a similar cognitive level [7], and allowing students themselves to learn the art of teaching. However, we believe that the choice to instrument a phenomenon occurring in a complex and potentially fragile system is not a trivial one. Student-led teaching already occurs in medical schools outside of the view of faculty members. Many educators are asking—How can we harness its potential? How can we incorporate it into the curriculum?—but, in our opinion, the question that comes before is—Should we?

A deep scholarly understanding of what occurs in peer-led teaching, including the motivation behind the individuals teaching, the nature of the interactions, and the learning processes involved must precede integration into formal curricular settings. An appreciation of the symbolism and meaning of teaching given by students to students, which occurs outside of faculty oversight, can ground such teaching in the culture of student practices. Such an exploration may be undertaken by qualitative studies such as ethnography or even peer ethnography, whereby members of the cohort to be studied are trained as researchers.

To us, the student ecosystem of education is a complex and multilayered system integrating formal and informal teaching from faculty members, peers, seniors, and outside resources. We believe that there is great pedagogical power in the peer-led learning aspect but also that it is precious. We suggest that before peer-led teaching is systematized, quantified, and introduced as a compulsory part of medical school, a deep exploration of givers and receivers of such education must be elicited. If not, faculty members may become part of the instrumentation which fundamentally alters an invisible system, casting the subatomic particle of peer teaching into an unknown trajectory.
